# Analysis of feline humeral fracture morphology and a comparison of fracture repair stabilisation methods: 101 cases (2009–2020)

**DOI:** 10.1177/1098612X221080600

**Published:** 2022-03-07

**Authors:** Nick Gall, Kevin Parsons, Heidi Radke, Eithne Comerford, Ben Mielke, James Grierson, John Ryan, Elena Addison, Vasileia Logethelou, Agnieszka Blaszyk, Sorrel J Langley-Hobbs

**Affiliations:** 1 Langford Veterinary Services, University of Bristol, Bristol, UK; 2 Department of Veterinary Medicine, University of Cambridge, Cambridge, UK; 3 School of Veterinary Science, Leahurst Campus, University of Liverpool, Liverpool, UK; 4 Department of Clinical Science and Services, Royal Veterinary College, London, UK; 5 Anderson Moores Veterinary Specialists, Winchester, UK; 6 Royal (Dick) School of Veterinary Studies and the Roslin Institute, The University of Edinburgh, Roslin, UK; 7 Division of Small Animal Clinical Science, School of Veterinary Medicine, University of Glasgow, Glasgow, UK

**Keywords:** Humeral fractures, orthopaedics, fracture repair, diaphyseal fractures, humerus, external skeletal fixator, fracture stabilisation, bone plating

## Abstract

**Objectives:**

The aims of this study were to describe the type, presentation and prognostic factors of feline humeral fractures over a 10-year period and to compare three stabilisation systems for feline humeral diaphyseal fractures.

**Methods:**

In total, 101 cats with humeral fractures presenting to seven UK referral centres between 2009 and 2020 were reviewed. Data collected included signalment, weight at the time of surgery, fracture aetiology, preoperative presentation, fixation method, surgical details, perioperative management and follow-up examinations. Of these cases, 57 cats with humeral diaphyseal fractures stabilised using three different fixation methods were compared, with outcome parameters including the time to radiographic healing, time to function and complication rate.

**Results:**

The majority of the fractures were diaphyseal (71%), with only 10% condylar. Of the known causes of fracture, road traffic accidents (RTAs) were the most common. Neutered males were over-represented in having a fracture caused by an RTA (*P* = 0.001) and diaphyseal fractures were significantly more likely to result from an RTA (*P* = 0.01). Body weight had a positive correlation (*r* = 0.398) with time to radiographic healing and time to acceptable function (*r* = 0.315), and was significant (*P* = 0.014 and *P* = 0.037, respectively). Of the 57 humeral diaphyseal fractures; 16 (28%) were stabilised using a plate–rod construct, 31 (54%) using external skeletal fixation and 10 (18%) using bone plating and screws only. Open diaphyseal fractures were associated with more minor complications (*P* = 0.048). There was a significant difference between fixation groups in terms of overall complication rate between groups (*P* = 0.012). There was no significant difference between fixation groups in time to radiographic union (*P* = 0.145) or time to acceptable function (*P* = 0.306).

**Conclusions and relevance:**

All three fixation systems were successful in healing a wide variety of humeral diaphyseal fractures. There was a significantly higher overall complication rate with external skeletal fixators compared with bone plating; however, the clinical impact of these is likely low.

## Introduction

Feline humeral fractures are relatively uncommonly encountered in veterinary practice, accounting for between 4.4% and 9.5% of feline fractures.^1–4^ Of these fractures, between 75% and 87% are diaphyseal;^4–6^ this is in contrast to dogs, which have a higher proportion of humeral condylar fractures.^4,5^ This difference is due to the relatively straight profile of the humeral shaft, the wider and straighter humeral condyles, and lack of supratrochlear foramen in the cat,^5–7^ and the well-documented prevalence of canine breed predisposition to structural weakness in the condylar area.^8–11^ Interestingly, a recent study identified 18 cats with suspected patellar fracture and dental anomaly syndrome^12^ that presented with humeral condylar fractures, six of which were bilateral and two demonstrated a humeral intercondylar fissure on the contralateral limb. This report suggested that there is likely an at-risk population of cats with a similar structural weakness to that identified in dogs.

Several reports have documented outcomes for various treatment methods for small numbers of feline humeral fractures, but no studies to date have compared the outcomes of different treatment options solely or exclusively in cats. Longley et al^13^ compared fixation methods in distal and supracondylar humeral fractures in a population of 12 cats and 25 dogs, which showed significantly higher rates of overall complications following use of external skeletal fixation (ESF) compared with plate and screw fixation but no with difference in final or long-term follow-up being reported in dogs and cats.

Intramedullary (IM) pinning with or without cerclage wire has been documented in feline humeral fracture repair in two small case series^14,15^ of 14 transverse or oblique fractures and one comminuted fracture. The average time to weight bearing was 25^14^ and 3–5^15^ days postoperatively, with no pin migration, bone shortening or fragment collapse radiographically evident in one study,^14^ and normal, complete fracture healing documented between 4 and 12 weeks in the other.^15^

The use of an interlocking nail (ILN) for feline humeral fracture repair was reported in one cat^16^ with a closed, grade V mid-diaphyseal humeral fracture that achieved radiographic union at 12 weeks postoperatively and was weightbearing with no lameness at 4 and 11 months after ILN placement. Another case series reported on five feline humeral fractures (a total of 121 diaphyseal fractures in dogs and cats) in which an ILN was used.^17^ Of these cases, 95% healed with good (favouring limb after exercise) or excellent (total absence of lameness) functional outcome and with 94% radiologically healed by 16 weeks. There was no mention of complications involving the feline humeral cases.

Bone plating (BP) has been documented as a fixation method for diaphyseal humeral fractures in dogs^18^ and approaches are described for cats.^19^ BP has also been reported in five cats with Y-T humeral fractures with supracondylar comminution.^20^ Three of five cases had a satisfactory outcome, with one being severely lame and the other requiring amputation due to implant failure. Plate–rod constructs (PRCs) have also been reported in humeral fracture fixation of cats. One study reported on minimally invasive plate osteosynthesis for PRC fixation in two cats with non-articular humeral fractures,^21^ with clinical union achieved at 36 ± 2 days with no complications and excellent functional outcome.

ESF was used to stabilise a variety of feline diaphyseal humeral fractures in a study of 13 cats.^22^ Eleven of 13 cats achieved union, with mean time to ESF removal in mildly comminuted fractures being 5 weeks and 4 days, and 10 weeks and 3 days in severely comminuted fractures. Linear–circular ESF has also been used for feline humeral fracture repair in two case series of four cats.^23,24^ Seven of eight cats in these studies had supracondylar or intracondylar humeral fractures. Seven of eight cats had excellent functional outcome; complication requiring revision surgery was only reported in one case.^24^

Therefore, the aims of this study were to detail the types of humeral fractures seen in cats at referral centres across the UK, and to compare the results of humeral diaphyseal fracture fixation based on radiographic and clinical findings, as well as report on the complications encountered with the different fixation methods. We hypothesised that fracture stabilisation with PRCs would result in a similar time to radiographic and clinical resolution as ESF stabilisation but have lower complication rates.

## Materials and methods

### Criteria of inclusion

Clinical records and radiographs of all cats presenting with humeral fractures to seven different referral centres around the UK between 2009 and 2020 were reviewed. Information collated included sex and neuter status, age and weight at the time of surgery, fracture aetiology, details of preoperative presentation, surgical fixation methods used, details of surgery, perioperative management and details of follow-up examinations.

Fractures were classified according to the level of comminution using a modified version of the Winquist Hansen system.^16,25^ Complications were classified as minor (that required no medical or surgical treatment to correct), major (surgical or medical treatment required for resolution) or catastrophic (permanent and unacceptable function of the limb, resulting in amputation).^26^ With regard to surgical fixation methods, for inclusion in the study, we required a full medical history with details of a humeral diaphyseal fracture repair using either ESF, PRCs or BP, information regarding the fixation method used and at least one follow-up examination with radiographs. When comparing the time taken to achieve radiographic union between the different fixation methods, cases that did not have radiographic union documented on follow-up radiographs were excluded.

### Surgical procedure

All surgeries were performed by board-certified surgeons or by residents in training under direct supervision of a board-certified surgeon. A craniolateral, lateral, craniomedial or minimally invasive approach was made for diaphyseal fractures dependent on surgeon preference, fixation system used and location of the fracture. For ESFs, either a type Ia, Ib, type II modified or I/II hybrid ESF was applied. An IM pin (1.6–2.4 mm) was used in all but one case and tied into the construct in two-thirds of cases. For ESF pins, primarily positive profile-end threaded half pins were used in the proximal areas of the humerus with a centrally threaded transcondylar pin being used when appropriate. All pins were placed according to established safe corridors of insertion^27^ and connected to the fixation bar with clamps.

Open reduction or minimally invasive approaches were used for plate fixation on the lateral, craniolateral, craniomedial or medial aspect of the humerus. Veterinary cuttable plate (2.0–2.7 mm; Depuy Synthes), locking compression plate (2 or 2.4 mm; B Braun Vetcare), dynamic compression plates (2.4 or 2.7 mm; Depuy Synthes) or string of pearls (Orthomed) plates (2 mm) were used, with bicortical screws being used where possible.

### Postoperative management

All owners were instructed to either crate or room rest their cats for a period of 4–8 weeks. Fractures were recorded as healed based on the surgeon’s case report referring to the radiographs. A delayed union was defined as fracture healing being evident but slower than the expected rate, but progressing to full union.^28^

Information was collected based on the reported findings from clinical examination by the veterinarian relating to complications, assessment of lameness and grade of function (unacceptable function, acceptable function or full function)^26^ of the operated limb from the case files. Postoperative re-checks were performed 4–8 weeks following the first recheck if necessary; recheck times were not standardised and at the surgeon’s discretion. Time to function was defined as the first entry of acceptable or full function of the operated limb in the records.

### Statistical analysis

Statistical analysis was performed using IBM SPSS Version 26.0. Surgical fixation methods were compared to ordinal variables using the Kruskal–Wallis test; when independent variables included only two groups, a Mann–Whitney U-test was used to assess against ordinal variables. Independent-scale variables were compared to ordinal variables or non-normally distributed scale variable using a Spearman rank correlation test. When two scale variables of normal distribution were compared, a simple linear regression test was used and when an ordinal independent variable was compared to a normally distributed dependent variable a one-way ANOVA was used. A *P* value of <0.05 was considered to be statistically significant.

### Ethical approval

Ethical approval for this study was granted by the Animal Welfare and Ethical Review Body (AWERB) on 22 September 2021 with a veterinary investigation number for reference of VIN/20/029.

## Results

A total of 101 feline humeral fractures were identified. The male to female ratio was 2:1 and a total of 11 breeds were represented, with 78% of the cases being domestic shorthair cats. Median age at presentation was 12 months (range 2–178) and mean body weight was 3.9 kg (range 800 g to 7 kg). Aetiology of the fracture was reported as being unknown in 47% (n = 47/101) of cases. Road traffic accidents (RTAs) were documented as the aetiology in 26% (n = 26/101) of cats. A summary of the fracture morphological characteristics and aetiologies are presented in Tables 1, 2 and 3.

**Table 1:** Proportion of cases in each sex and neuter status group that were confirmed to be caused by an road traffic accident (RTA)

Cause of fracture	MN	ME	FN	FE	Total
RTA	17 (65.4)	1 (3.8)	7 (26.9)	1 (3.8)	26 (100.0)
Other	9 (32.1)	9 (32.1)	5 (17.9)	5 (17.9)	28 (100.0)
NR	21 (44.7)	4 (8.5)	11 (23.4)	1 (2.1)	37 (100.0)

Data are n (%)

MN = male neutered; ME = male entire; FN = female neutered; FE = feline entire; NR = not reported

**Table 2:** Fracture characteristics and variation between cats in our study

Fracture characteristics		Cats in the present study	Cases in the canine population^4,5^
Open or closed	Open	11 (12)	–
	Closed	80 (88)	–
Fracture grade	0	21 (23)	–
	1	9 (10)	–
	2	15 (16)	–
	3	23 (26)	–
	4	18 (20)	–
	5	4 (4)	–
Fracture orientation	Oblique	27 (30)	9/42 (21)^5^
	Comminuted	49 (55)	15/42 (36)^5^
	Transverse	5 (6)	12/42 (29)^5^
	Spiral	8 (9)	4/42 (10)^5^
Fracture position	Diaphyseal	65 (71)	42/107 (39),^5^ 6/22 (27)^4^
	Supracondylar	12 (13)	16/107 (15)^5^
	Condylar	9 (10)	43/107 (40),^5^ 16/22 (73)^4^
	Articular	4 (4)	–
	Physeal	1 (1)	5/107 (5%),^5^ 1/22 (5%)^4^

Data are n (%)

**Table 3:** Distribution of fracture positions between cases confirmed to be caused by a road traffic accident (RTA) or not and a summary of the aetiologies for each fracture position

	Fracture position					Total
Cause of fracture	Diaphyseal	Supracondylar	Condylar	Articular	Physeal	
RTA	23	1	0	2	0	26
Other	15 (other trauma [n = 6], gunshot [n = 4], fall from height [n = 3], dog attack [n = 2])	4 (object landing on cat [n = 2], fall from height [n = 1], gunshot [n = 1])	6 (pathological [n = 3], fall [n = 2], object falling on cat [n = 1])	2 (fall [n = 2])	1 (fall [n = 1])	28
Total	38	5	6	4	1	

Of the 57 humeral diaphyseal fractures reviewed, 16 (28%) were stabilised using a PRC, 31 (54%) using ESF and 10 (18%) using BP. There was no significant difference between treatment groups in terms of age, weight, fracture grade or whether a fracture was open or closed.

All cats underwent general anaesthesia for surgery supervised by a veterinary anaesthetist; local analgesic nerve blocks were performed at their discretion. A summary of perioperative and postoperative management can be found in Tables 4 and 5.

**Table 4:** Details of intraoperative antibiotics and postoperative antibiotic administration in each diaphyseal fracture stabilisation group (where available, the doses and frequency of administration are included)

	Perioperative IV antibiotics	Frequency of intraoperative antibiotics	Postoperative antibiotics (yes/no)	Type of postoperative antibiotics
PRC	Cefuroxime 20 mg/kg (14/16) Amoxicillin–clavulanate 20 mg/kg (2/16)	q90mins (11/16) q120mins (5/16)	Yes (10/16) No (6/16)	Cephalexin 15–20 mg/kg 5–14 days (6/10) Amoxicillin–clavulanate 14 days (2/10) Amoxicillin–clavulanate/enrofloxacin 14 days (1/10) Cefovecin SC (1/10)
BP	Amoxicillin–clavulanate 20 mg/kg (1/9)	q90mins or less (3/9) q120mins (6/9)	Yes (6/9) No (3/9)	Cephalexin 7 days (6/6)
ESF	Cefuroxime 20 mg/kg (16/24) Amoxicillin–clavulanate 15–20 mg/kg (7/24) Ceftiofur (1/24)	q90mins or less (14/24) q120mins or more (10/24)	Yes (18/26) No (8/26)	Cephalexin 15–20 mg/kg 5–10 days (12/18) Amoxicillin-clavulanate 15–20 mg/kg 10–14 days (3/18) Unspecified antibiotics (3/18)

IV = intravenous; PRC = plate–rod construct; SC = subcutaneously; BP = bone plating; ESF = external skeletal fixation

**Table 5:** Number of patients discharged with oral analgesia

	Oral analgesia at discharge* (yes/no)	Description of analgesia
PRC	Yes (16/16) No (0/16)	Meloxicam 0.05 mg/kg PO (16/16)
BP	Yes (9/9) No (0/9)	Meloxicam 0.05 mg/kg PO (8/9) Buprenorphine 0.02 mg/kg PO (1/9)
ESF	Yes (23/24) No (1/24)	Meloxicam 0.05 mg/kg PO (23/23)

Data were not collected on the immediate postoperative analgesia regime

* All patients given intravenous analgesia immediately after surgery and transitioned onto oral analgesia over the subsequent days

PRC = plate–rod construct; BP = bone plating; ESF = external skeletal fixation

**Figure 1:**
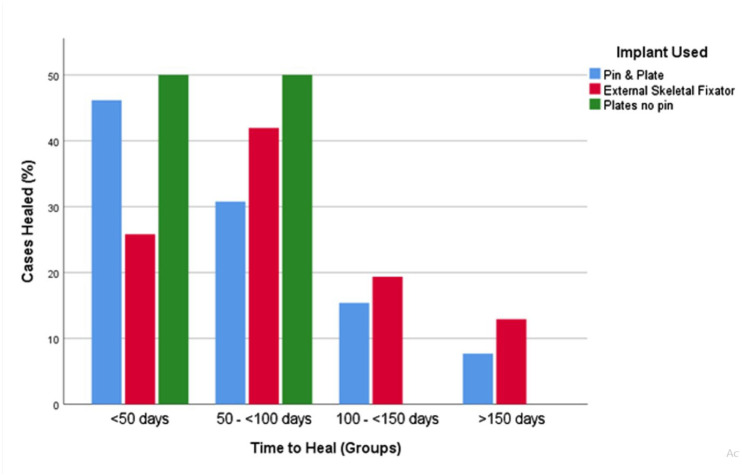
Time taken for the cases within each diaphyseal fracture treatment group to radiographically heal. The x-axis is divided into 50-day increments and the percentage of cases healed from each group within this time are displayed. Fractures that did not have documented union are not included in this figure

Open fractures represented 18% (9/49) of fixation cases, with the remaining 82% (40/49) being closed. Median time to healing in the BP group was 53 days (range 28–98), and it was 56 days (range 28–154) in the PRC group and 70 days (range 21–448) in the ESF group (Figure 1). Median time to function in the BP group was 42 days (range 28–77), and it was 53 days (range 28–70) in the PRC group and 49 days (range 21–182) in the ESF group. A total of 3/57 (5%) fractures did not have documented union and had delayed healing reported on their radiographs. All three of these occurred in the PRC group, giving an 81% (13/16) documented healing rate vs 100% (31/31 and 10/10, respectively) achieving union in the ESF and BP groups. A summary of the cases that did not have documented radiographic union can be found in Table 6. The overall complication rates were 10% (1/10), 50% (8/16) and 65% (20/31) in the BP, PRC and ESF groups, respectively. Details of the complications can be found in Table 7.

**Table 6:** Descriptions of the three cases in the plate–rod construct group that did not have documented radiographic union after fracture repair and the reported radiographic findings from their first recheck after surgery

Signalment	Fracture description	Radiographic appearance at first recheck
6-year-old FN DSH aged 6 years	Grade IV, closed, mid-diaphyseal fracture	Some remodelling but no obvious callous at 6 and 9 weeks, lost to follow-up
FE DSH aged 14 years 9 months	Grade 0, closed, mid-to-distal oblique diaphyseal fracture	Delayed healing at 7 weeks, lost to follow-up
MN DSH aged 6 years 6 months	Grade II, closed, oblique mid-diaphyseal fracture	Implant failure, amputation performed

FN = female neutered; DSH = domestic shorthair; FE = female entire; MN = male neutered

**Table 7:** Complications encountered in each diaphyseal fracture treatment group, divided into catastrophic, major or minor, with a description of the complications encountered

Fixation group	Complication (description)		
	Catastrophic	Major	Minor
BP	0	1 (fracture misaligned and required revision surgery the following day)	0
PRC	1 (implant failure necessitating amputation)	3 (IM pin migration necessitating removal under general anaesthesia [n = 2], screws pulling out requiring repeat surgery to correct [n = 2])	5 (single screw pulling out not needing surgical intervention [n = 2], muscular atrophy [n = 2] and delayed healing not requiring further treatment [n = 1])
ESF	0	12 (infection of one of the pins requiring antibiotics or implant removal [n = 7], repeat surgery to place a bone graft or modification of the fixation due to delayed healing [n = 3], intra-articular pin placement [n = 1] and refracture of the humerus 1 month following ESF removal necessitating repeat surgery [n = 1])	11 (superficial infection of a pin tract not needing antibiotic therapy [n = 8], delayed healing not requiring treatment [n = 1], reduced ROM in elbow [n = 1] and elbow subluxation that resolved following planned ESF removal [n = 1])

BP = bone plating; PRC = plate–rod construct; IM = intramedullary; ESF = external skeletal fixation; ROM = range of motion

There was a significant difference between the sexes and whether the fracture was caused by an RTA or not (*P* = 0.001), with neutered males being over-represented in the RTA group. There was also a significant difference between fracture position and aetiology of the fracture (*P* = 0.01), with significantly more diaphyseal fractures being caused by RTAs. The aetiology of the fracture had no significant effect on fracture grade (*P* = 0.160) but did have a significant effect on whether a fracture was open or closed (*P* < 0.001).

The age of the cat had no significant correlation to time to radiographic union (*P* = 0.1) or complication rate (*P* = 0.371) when comparing the fracture stabilisation method used. However, increasing body weight had a positive correlation (*r* = 0.398) with time to radiographic healing and time to acceptable function (*r* = 0.315), which was significant (*P* = 0.014 and *P* = 0.037, respectively) but had no association with complication rate (*P* = 0.6).

Fracture grade had no statistically significant effect on the time to healing (*P* = 0.641), time to function (*P* = 0.427) or the overall complication rate (*P* = 0.592).

Whether a fracture was open or closed had no significant effect on the time taken to heal (*P* = 0.155), time to acceptable function (*P* = 0.195) or overall complication rate (*P* = 0.104). However, open fractures had significantly more minor complications than closed fractures (*P* = 0.048).

There was no significant difference in time to radiographic union between the fixation groups (*P* = 0.196); however, the difference in documented time to heal was significant (*P* = 0.019). The rate of complications was significantly different when comparing the method of repair (*P* = 0.012); however, implant selection had no significant effect on catastrophic (*P* = 0.322), major (*P* = 0.181) or minor (*P* = 0.113) complications.

## Discussion

Diaphyseal humeral fractures were the most common fracture types in our population of cats, representing 71% of the fractures vs 27–39%^4,5^ previously reported for dogs. Condylar fractures were relatively uncommon, representing only 10% of fractures vs 40–73%^4,5^ reported in canine studies. This study demonstrated that ESF, PRC and BP were all successful methods of stabilisation for a wide variety of humeral diaphyseal fractures. Although ESF was the only device used for the most severely comminuted fractures (grade V), there was no significant difference in fracture grade, open/closed fractures or fracture orientation between implant groups. Our findings suggest that there is no advantage when selecting a particular fixation system for a type of humeral fracture so the surgeon should choose the system they think is most appropriate for the fracture according to their preference and experience.

The most common aetiology for humeral fracture encountered in this population was an RTA. However, this figure is very likely an underestimate as the cause of trauma was not clearly identified in nearly half of the cats. In many of these, an RTA can be presumed. Male cats were significantly more likely to be involved in RTAs than female cats, which has also been found as a risk factor in previous studies^29–31^ and is suspected to be related to the differing roaming or behavioural habits of male cats, although evidence for this is conflicting.^31–34^ Other risk factors for RTAs,^30,31^ such as age and breed, were not found to have a significant association with RTAs in this study.

There was a significant association between diaphyseal fractures and RTAs. This may be due to cats most often being hit perpendicular to the long axis of the humerus and the shaft experiencing supraphysiological bending and shear forces upon it. Although RTAs are considered to be high-impact traumas, there was no association between the level of comminution and whether the fracture was caused by an RTA or not.

There were no significant differences in healing time between implant groups. Variability between recheck was an anticipated limitation of our retrospective data collection, and significance may have been found with more standardised recheck times. All cases that did not have documented union were in the PRC group. It is likely that the cases did in fact achieve union but the time that this occurred could not be documented as they did not return for further rechecks. The positive correlation of body weight with time to healing was also documented in feline femoral fractures,^35^ and it was hypothesised that heavier cats have a prolonged healing time, which could be due to the higher forces imparted on the bones following fracture fixation. Larger implants are generally used in heavier cats, however, which should negate this increased force. Further research is required into this, and body condition score should also be recorded in future studies.

Open fractures were not common and were significantly more likely to be caused by gunshot wounds than other traumas; all of the fractures caused by gunshots were open. A previous study not only found RTAs and other high-velocity traumas to be risk factors for open fractures in cats, but also that the humerus is one of the lowest-risk bones for open fractures in the appendicular skeleton.^36^ Minor complications were significantly higher in cats with open fractures compared with those with closed fractures. Most of the minor complications found in our study involved infection or delayed healing, which could be associated with the open nature of the original injury.^37^ The retrospective nature of this study limited the detail to which open fractures were described. A more comprehensive classification system such as the Gustilo-Anderson Open Fracture Classification Scheme,^37,38^ or the new scheme proposed by the Orthopaedic Trauma Association,^39^ could be used in future studies to allow more accurate analysis of open fractures and their effect on the complication rate and clinical outcome.

There was a significant difference between fixation methods in the overall complication rate. The majority of the complications associated with the ESF group were pin tract infections. It is important to note that minor pin tract infections are almost inevitable with ESF due to soft tissue impalement and motion of soft tissue around the pin.^6,40^ A previous study showed pin tract infections in feline humeral ESF to be exclusively superficial, rather than deep infections, and manageable.^41^ An alternative method for future prospective studies could include the use of implant–skin interface scoring system for ESF.^42^

The findings of this humeral fracture study are similar to those found in a previous study comparing feline femoral diaphyseal fracture stabilisation.^35^ There were no significant differences in time to radiographic union between the BP, ESF and PRC groups when they were used to stabilise feline femoral diaphyseal fractures and ESF also had the highest number of minor complications in feline femoral fractures. PRCs had the least complications in feline femoral diaphyseal fracture fixation, whereas BP showed the lowest complication rate in this study. PRCs were used in more than half of the cases in the femoral fracture fixation study but only a quarter of the humeral fracture cases. The lack of medullary canal distal to the epicondylar ridge in many cat humeri has been shown to make IM pin placement more challenging in this bone,^27^ which may have contributed to the comparatively higher complication rate seen in PRC humeral fracture repair.

This was a multicentre study and so had the inevitable limitations associated with that including different surgeons, protocols and variable follow-up times. Owing to the relatively low incidence of feline humeral fractures seen each year in referral practice, it would take a long time to perform a prospective single-centre study comparing outcomes from feline humeral fracture fixation. There were differing numbers of cases for each fixation group, and having similar numbers for each stabilisation system would have been optimal for data analysis.

## Conclusions

Diaphyseal fractures were the most common humeral fracture type seen in cats. PRCs, BP and ESF were all applied successfully for the stabilisation of such fractures. ESF was associated with a higher number of complications; however, the majority of these complications were manageable and had no bearing on overall outcome.

## References

[1] Cardoso CB Rahal SC Agostinho FS , et al. Long bone fractures in cats: a retrospective study. Vet Zoo 2016; 23: 504–509.

[2] Hill FWG . A survey of bone fractures in the cat. J Small Anim Pract 1977; 18: 457–463. 886835 10.1111/j.1748-5827.1977.tb05912.x

[3] Ness MG Abercromby RH May C , et al. A survey of orthopaedic conditions in small animal veterinary practice in Britain. Vet Comp Orthop Traumatol 1996; 9: 43–52.

[4] Phillips IR . A survey of long bone fractures in dogs and cats. J Small Anim Pract 1979; 20: 661–674. 547112 10.1111/j.1748-5827.1979.tb06679.x

[5] Bardet JF Hohn RB Rudy RL , et al. Fractures of the humerus in cats and dogs: a retrospective study of 130 cases. Vet Surg 1983; 12: 73–77.

[6] Langley-Hobbs SJ . Fractures of the humerus. In: Tobias KM Johnston SA (eds). Veterinary surgery: small animal. 2nd ed. New York: Elsevier Health Sciences, 2017, p 821.

[7] Scott HW McLaughlin R . The humerus. In: Feline orthopaedics. 2nd ed. Boca Raton, FL: CRC Press, 2006, p 126.

[8] Vilamil CS Phillips ASJ Pegram CL , et al. Impact of breed on canine humeral condylar fracture configuration, surgical management and outcome. Vet Surg 2020; 49: 639–647. 32311154 10.1111/vsu.13432

[9] Denny HR . Condylar fracture in the humerus of the dog: a review of 133 cases. J Small Anim Vet Pract 1983; 24: 185–197.

[10] Rørvik AM . Risk factors for humeral condylar fractures in the dog: a retrospective study. J Small Anim Pract 1993; 34: 277–282.

[11] Marcellin-Little DJ DeYoung DJ Ferris KK , et al. Incomplete ossification of the humeral condyle in Spaniels. Vet Surg 1994; 23: 475–487. 7871711 10.1111/j.1532-950x.1994.tb00509.x

[12] Chan AJH Reyes Rodriguez NA Bailey SJ , et al. Treatment of humeral condylar fractures and humeral intracondylar fissures in cats with patellar fracture and dental anomaly syndrome. J Feline Med Surg 2020; 22: 1008–1015. 32129129 10.1177/1098612X20904458 PMC7521005

[13] Longley M Chase D Calvo I , et al. A comparison of fixation methods for supracondylar and distal humeral shaft fractures of the dog and cat. Can Vet J 2018; 59: 1299–1304. 30532287 PMC6237257

[14] Ozsoy S . Fixation of femur, humerus and tibia fractures in cats using intramedullary threaded Steinmann pins. Vet Rec 2004; 155: 152–153. 15338710 10.1136/vr.155.5.152

[15] Altunatmaz K Ozsoy S Mutlu Z , et al. Use of intramedullary fully threaded pins in the fixation of feline and canine humeral, femoral and tibial fractures. Vet Comp Orthop Traumatol 2012; 25: 321–325. 22580851 10.3415/VCOT-11-05-0068

[16] Moses PA Lewis DD Lanz OI , et al. Intramedullary interlocking nail stabilisation of 21 humeral fractures in 19 dogs and one cat. Aust Vet J 2002; 80: 336–343. 12153056 10.1111/j.1751-0813.2002.tb14781.x

[17] Duhautois B . Use of veterinary interlocking nails for diaphyseal fractures in dogs and cats: 121 cases. Vet Surg 2003; 32: 8–20. 12520485 10.1053/jvet.2003.50008

[18] Harari J Roe SC Johnson AL . Medial plating for repair of middle and distal diaphyseal fractures of the humerus of the dog. Vet Surg 1986; 15: 45–48.

[19] Schmierer PA Pozzi A . Guidelines for surgical approaches for minimally invasive plate osteosynthesis in cats. Vet Comp Orthop Traumatol 2017; 30: 272–278. 28636054 10.3415/VCOT-16-07-0105

[20] Macias C Gibbons SE McKee WM . Y-T fractures with supracondylar comminution in five cats. J Small Anim Pract 2006; 47: 89–93. 16438696 10.1111/j.1748-5827.2006.00026.x

[21] Guiot LP Guillou RP Dejardin LM . Minimally invasive percutaneous medial plate–rod osteosynthesis for treatment of humeral shaft fractures in dog and cats: surgical technique and prospective evaluation. Vet Surg 2019; 48(S1): O41–O51. 30444262 10.1111/vsu.13134

[22] Langley-Hobbs SJ Carmichael S McCartney WT . External skeletal fixation for stabilisation of comminuted humeral fractures in cats. J Small Anim Pract 1997; 38: 280–285. 9239628 10.1111/j.1748-5827.1997.tb03465.x

[23] Silva HR Clements DN Yeadon R , et al. Linear-circular external skeletal fixation of intracondylar humeral fractures with suracondylar comminution in four cats. Vet Comp Orthop Traumatol 2012; 25: 61–66. 22027756 10.3415/VCOT-11-04-0059

[24] Kirkby KA Lewis DD Lafuente MP , et al. Management of humeral and femoral fractures in cats and dogs with linear-circular hybrid external skeletal fixators. J Am Anim Hosp Assoc 2008; 44: 180–197. 18593855 10.5326/0440180

[25] Winquist RA Hansen ST Jr . Comminuted fractures of the femoral shaft treated by intramedullary nailing. Orthop Clin North Am 1980; 11: 633–648. 7413179

[26] Cook JL Evans R Conzemius MG , et al. Proposed definitions and criteria for reporting time frame, outcome and complications for clinical orthopaedic studies in veterinary medicine. Vet Surg 2010; 39: 905–908. 21133952 10.1111/j.1532-950X.2010.00763.x

[27] Langley-Hobbs SJ Straw M . The feline humerus. An anatomical study with relevance to external skeletal fixator and intramedullary pin placement. Vet Comp Orthop Traumatol 2005; 18: 1–6. 16594209

[28] Crenshaw A . Delayed union and non-union of fractures. In: Crenshaw A (ed). Campbell’s operative orthopaedics, Vol 3. St Louis, MO: CV Mosby, 1987, p 118.

[29] Conroy M O’Neill D Boag A , et al. Epidemiology of road traffic accidents in cats attending emergency care practices in the UK. J Small Anim Pract 2019; 60: 146–152. 30383291 10.1111/jsap.12941

[30] Rochlitz I . Study of factors that may predispose domestic cats to road traffic accidents: part 1. Vet Rec 2003; 153: 549–553. 14627234 10.1136/vr.153.18.549

[31] Rochlitz I . Study of factors that may predispose domestic cats to road traffic accidents: part 2. Vet Rec 2003; 153: 585–588. 14640325 10.1136/vr.153.19.585

[32] Rochlitz I . A review of the housing requirements of domestic cats (Felis silvestris catus) kept in the home. Appl Anim Behav Sci 2005; 93: 97–109.

[33] Barratt DG . Home range size, habitat utilisation and movement patterns of suburban and farm cats *Felis catus*. Ecography 1997; 20: 271–280.

[34] Liberg O Sandell M Pontier D , et al. Density, spatial organisation and reproductive tactics in the domestic cat and other fields. In: Turner D Bateson P (eds). The domestic cat: the biology of its behaviour. 3rd ed. Cambridge: Cambridge University Press, 2013, pp 119–147.

[35] Konning T Maarschalkerweerd RJ Endenburg N , et al. A comparison between fixation methods of femoral diaphyseal fractures in cats – a retrospective study. J Small Anim Pract 2013; 54: 248–252. 23560936 10.1111/jsap.12061

[36] Millard RP Weng HY . Proportion of and risk factors for open fractures of the appendicular skeleton in dogs and cats. J Am Vet Med Assoc 2014; 245: 663–668. 25181270 10.2460/javma.245.6.663

[37] Gustilo RB Mendoza RM Williams DN . Problems in the management of type III (severe) open fractures: a new classification of type III open fractures. J Trauma 1984; 24: 742–746. 6471139 10.1097/00005373-198408000-00009

[38] Gustilo RB Anderson JT . Prevention of infection in the treatment of one thousand and twenty-five open fractures of long bones: retrospective and prospective analyses. J Bone Joint Surg 1976; 58: 453–458. 773941

[39] Orthopaedic Trauma Association: Open Fracture Study Group. A new classification scheme for open fractures. J Orthop Trauma 2010; 24: 457–464. 20657245 10.1097/BOT.0b013e3181c7cb6b

[40] Jaeger GH Wosar MA . External skeletal fixation. In: Tobias KM Johnston SA (eds). Veterinary surgery: small animal. 2nd ed. New York: Elsevier Health Sciences, 2017, p 720.

[41] Beever L Giles K Meeson R . Postoperative complications associated with external skeletal fixators in cats. J Feline Med Surg 2017; 19: 727–736. 28592224 10.1177/1098612X17699466 PMC11129199

[42] McDonald-Lynch MB Marcellin-Little DJ Roe SC , et al. Assessment of an implant-skin interface scoring system for external skeletal fixation of dogs. Am J Vet Res 2015; 76: 931–938. 26512537 10.2460/ajvr.76.11.931

